# Address of C. K. Winston, M. D.

**Published:** 1857-06

**Authors:** 


*Address of* C. K. Winston, M. D., *Chairman of the Committee of Arrangements, for the American Medical Association, on behalf of the Committee, and of the Medical Profession of Nashville.*

Mr. President and Gentlemen of the American Medical Association :—This, I believe, is the Tenth Annual meeting of this Association. As chairman of the Committee of Arrangements and Reception, I am charged with the agreeable duty of welcoming you to the State of Tennessee and the City of Nashville. I regret that I have not language to express this sentiment with sufficient cordiality. I only add, gentlemen, in common phrase, " You are more than welcome."

You are the representatives of a profession, distinguished alike for its antiquity, its scientific attainments, and its usefulness. It constitutes the true link between science and philanthropy--moral, intellectual and physical. You come from every portion of this glorious republic--from the Kenebec to the Rio Grande--from orange groves and golden sands--from mountains clad in eternal snow, and valleys smiling in perpetual verdure. You come not for purposes of self aggrandizement or personal ambition, nor yet to advance the schemes of parties or stir up the antipathies of sections. "You know no North, no South, no East, no West;" but you come as a company of philanthropists, a band of brethren, that you may pour the acquisitions of another year into a common treasury, kneel side by side at a common altar, and drink the living water as it gushes from a common fountain. You have come to maintain the dignity, to elevate the ensign of a profession, to which you have devoted your lives, and to which you have linked your fortunes.

You are the cultivators of a profession eminently progressive, admitting to the fullest extent the spirit and genius of enterprise. So much may not be so fully said of others. Who could expect at this or any other day, to embellish the Commentaries of Blackstone, or improve the pleadings of Chitty, or re-poise the scales of justice? Where are the men with commissions never so divine, who would attempt to re-cast the logic which made Felix tremble, or adorn the doctrine of justification by faith? Who hopes now to shed additional light on the pathway to the skies, or sing in strains more immortal than the triumphs of the cross? Not so with Medicine. Yours is a rising orb—magnificent in its proportions—while others have reached the zenith, yours has but begun to mount the heavens--while others have begun to fade, yours knows no eclipse nor decline. You revere the names of Hippocrates and Sydenham, of Brown and Cullen, with a host of others; you treasure up their maxims, but you acknowledge no *master*, you fall down at the feet of no *Gamaliel.* You have come to the day of free thought, of free investigation and free speech. You call in question the most hoary, as well as the most recent fact, and you are daily revealing, in floods of light, principles hid from the foundation of the world.

You are eminently the students of nature. While others may be led along dubious by mortal pedagogues, your teacher dwells in the realms of eternel light, and guides with hand unseen and unerring to essences and first causes. The formative chrystal and germinal dot are alike transparent before you. You are taught the mysteries of the living principle; the scalpel and retort are your companions, while you revel in the wonders of the microscopic world. You understand, somewhat, the laws by which a mote or a mountain is formed, a monad or a man is m[illegible]. The spear of grass which lifts its head in the distant solitude, the lordly oak and imperial ceder, instruct you, while air and earth, and sea, with the creeping multitude yield treasures at your command.

You are the veterans of a thousand battle fields, not in mortal strife where man meets man in sanguinary conflict; but where a secret and impalpable foe—a tyrant who has reigned from Adam till now—disposes his secret forces and directs their deadly shafts. When others have turned back affrighted and aghast, you, single-handed and alone, have met "the pestilence which walketh in the darkness," and the destruction "which wasteth at noonday," despoileth them of "the armor wherein they trusted," and have driven them ignominious, from the field.

Were the victories which you have won, the conquests which you have achieved known, you would be crowned with laurels more unfading than those which entwined the brows of Greek or Roman conquerers.

But more and better than all, you are the lovers of your race, the friends of humanity. Scattered about all over this happy land, you emphatically "go about doing good." Your hearts beat in unison with human woe—your ears are open to the cry of distress, whether it come from hovel or palace—you "wipe away the orphan's tear and cause the widow's heart to sing for joy,"—upon your heads daily descend "the blessings of those who were ready to perish."

To such a body of men, thus actuated, thus coming, we extend a cordial welcome. We feel honored by your presence, and expect to be improved and elevated by your intercourse. We throw wide our doors and invite you to the hospitalities of our homes, and to the kindred affections of our hearts.—*Nashville Medical & Surgical Journal.*

